# Fetal dose minimization: ultra-low dose long axial field of view (LAFOV) PET/CT imaging of a pregnant patient

**DOI:** 10.1007/s00259-024-06861-0

**Published:** 2024-08-03

**Authors:** J. H. van Snick, K. P. Koopmans, A. W. J. M. Glaudemans, G. N. Stormezand, O. V. Ivashchenko

**Affiliations:** Department of Nuclear Medicine and Molecular Imaging, University Medical Center Groningen, Hanzeplein 1, Groningen, GZ, 9713 The Netherlands

A 29-year-old patient, weighing 60 kg, was referred for [18 F]FDG PET/CT imaging at 18 weeks of pregnancy, a critical stage for fetal development with increased sensitivity to radiation [1]. This scan was part of the eligibility screening for external beam radiotherapy treatment for Hodgkin lymphoma IVA.

Utilizing the ultra-high-sensitivity mode of the Biograph Vision Quadra system (Siemens Healthineers) [2], we performed a 15-minute head-to-mid-femur scan (60 min post-injection). This was reconstructed into shorter scan durations to determine an acceptable duration using Likert scores and feedback from three nuclear medicine physicians. To minimize fetal exposure, only 20 MBq of [18 F]FDG was administered, and a tin filter was used to reduce CT dose (Sn140, 17 mAs, 1.7 pitch, CTDIvol = 0.4 mGy).

The effective fetal dose was quantified at 0.13 mSv for internal (PET) and 0.58 mSv for external radiation (CT) [3, 4], approximately 140 times below thresholds known to affect cognitive development and recommended levels for minimizing fetal exposure [5].



The 5-minute scan was identified as the shortest duration with excellent clinical image quality. Pathologic uptake in enlarged supraclavicular, mediastinal, and intra-abdominal lymph nodes was clearly visible, along with small pulmonary lesions in the right lung (4–14 mm). Pregnancy-related changes, such as increased mammary uptake and increased activity in the ovaries and uterus, were evident, along with traces of fetal [18 F]FDG uptake.

This case demonstrates the effectiveness of LAFOV PET/CT imaging in pregnant patients, minimizing fetal radiation exposure while maintaining high-quality maternal imaging. Other low-dose applications include patient groups with vulnerable genomes, children, and regular follow-ups.

## Data Availability

The datasets generated during and/or analyzed during the current study are available from the corresponding author on reasonable request.
